# Advanced Kidney Models In Vitro Using the Established Cell Line Renal Proximal Tubular Epithelial/Telomerase Reverse Transcriptase1 for Nephrotoxicity Assays

**DOI:** 10.3390/biomimetics9070446

**Published:** 2024-07-22

**Authors:** Alodia Lacueva-Aparicio, Laura Martínez-Gimeno, Pilar Torcal, Ignacio Ochoa, Ignacio Giménez

**Affiliations:** 1 Renal and Cardiovascular Physiopathology (FISIOPREN), Aragon Health Science Institute, 50009 Zaragoza, Spainigimenez@unizar.es (I.G.); 2 Tissue Microenvironment Lab (TME Lab), I3A, University of Zaragoza, 50018 Zaragoza, Spain; iochgar@unizar.es; 3 Institute for Health Sciences of Aragon (IACS), 50009 Zaragoza, Spain; 4 Aragón Health Research Institute (IISAragón), 50009 Zaragoza, Spain; 5 School of Medicine, University of Zaragoza, 50009 Zaragoza, Spain; 6 CIBER in Bioengineering, Biomaterials and Nanomedicine (CIBER-BBN), 28029 Madrid, Spain

**Keywords:** kidney, in vitro models, kidney-on-a-chip, 3D structures, hydrogel, nephrotoxicity

## Abstract

Nephrotoxicity stands as one of the most limiting effects in the development and validation of new drugs. The kidney, among the organs evaluated in toxicity assessments, has a higher susceptibility, with nephrotoxic potential frequently evading detection until late in clinical trials. Traditional cell culture, which has been widely used for decades, does not recapitulate the structure and complexity of the native tissue, which can affect cell function, and the response to cytotoxins does not resemble what occurs in the kidney. In the current study, we aimed to address these challenges by creating in vitro kidney models that faithfully biomimic the dynamics of the renal proximal tubule, using the well-established RPTEC/TERT1 cell line. For doing so, two models were developed, one recreating tubule-like structures (2.5D model) and the other using microfluidic technology (kidney-on-a-chip). The 2.5D model allowed tubular structures to be generated in the absence of hydrogels, and the kidney-on-a-chip model allowed shear stress to be applied to the cell culture, which is a physiological stimulus in the renal tissue. After characterization of both models, different nephrotoxic compounds such as cisplatin, tacrolimus, and daunorubicin were used to study cell responses after treatment. The developed models in our study could be a valuable tool for pre-clinical nephrotoxic testing of drugs and new compounds.

## 1. Introduction

The kidney is one of the major targets in terms of drug-induced toxicity in the human body. Predicting nephrotoxicity in preclinical drug development is challenging, and the potential for kidney toxicity in newly developed drugs is frequently underestimated. Most of the drugs that produce nephrotoxicity in the clinical phase do not show the same effects in the preclinical phase [1]. There is still not an in vitro model of the kidney that is ideal for the study of drug nephrotoxicity, but great progress is being made in this regard. New in vitro models are being developed considering the three-dimensional structure of the kidney, the components of its extracellular matrix (ECM), the shear stress to which the cells are subjected, and the combination of different cell types. All those characteristics could help predict potential kidney damage that might result from exposure to certain drugs [2,3,4].

Several sophisticated kidney models have been used for nephrotoxicity testing. In vitro models based on recreating tubule-like structures, recellularization of the kidney extracellular matrix, generation of renal spheroids, and kidney-on-a-chip models have been used for this purpose [5,6,7,8,9,10,11,12]. These models have not been universally implemented for pre-clinical phases of drug development for several reasons. These models are difficult to apply on a large scale for drug screening due to their complexity, such as the organ-on-a-chip-based models, as most of the studies using this technology do not use commercial devices but custom-made ones, which limits their use [13,14,15,16].

Models that make use of extracellular matrix proteins to recreate structures like those found in vivo also have their limitations. The cells in these models are often embedded within hydrogels and are difficult to access, making drug treatments difficult to perform [17,18]. Progress is currently being made in the recreation of three-dimensional structures from embryonic or adult stem cells to generate kidney organoids, with the aim of creating more complex structures, as the kidney is a very complex organ with diverse cell types [19,20,21,22]. These models have the advantage of being a more reliable representation of renal tissue due to their complexity, but they also have their limitations, such as the fact that they are time-consuming and costly protocols, so drug screening with organoids is still a goal to be achieved [23,24].

In the present work, we have developed two different models based on available commercial devices and equipment, taking advantage of our improved user-friendly methodology to perform complete kinetic studies within a single device. One of these models is based on the recreation of tubular structures using proteins from the extracellular matrix. The second model is based on kidney-on-a-chip models perfusing cell culture medium to apply shear stress to the cell culture. These models were compared to traditional cell culture when cells were exposed to different known nephrotoxic compounds such as cisplatin, daunorubicin, and tacrolimus.

## 2. Materials and Methods

### 2.1. Chemicals and Solutions

Cisplatin was prepared in a 0.9% NaCl solution in distilled water at a concentration of 3 mM and stored at 4 °C. Tacrolimus, was diluted in DMSO to a concentration of 24.33 mM and stored at −20 °C. Daunorubicin was prepared in distilled water at a concentration of 50 mM and stored at −20 °C. All compounds were purchased from Sigma-Aldrich.

### 2.2. Cell Culture

Human renal proximal tubular epithelial cell line RPTEC/TERT1 (Evercyte GmbH, Vienna, Austria) was cultured in a mix of DMEM-F12 w/o glutamine, w/o HEPES (BioWest, Nuaillé, France, L0090) and DMEM-F12 w/o glutamine, w/o HEPES, w/o glucose (BioWest, Nuaillé, France, L0091) containing 5 mM glucose final concentration and supplemented with 10 mM HEPES (Biochrom, Cambridge, UK, AGL1613), 2 mM GlutaMax (Gibco, Dublin, Ireland, 35050-038), hEGF 10 ng/mL (Sigma E9644), T3 5 pM (Sigma T6397), L-ascorbic acid 3.5 µg/mL (Sigma A4544), Transferrin Holo 5 µg/mL (Merck 616424), prostaglandin E1 25 ng/mL (Sigma P8908), hydrocortisone 25 ng/mL (Sigma H0396), sodium selenite 8.65 ng/mL (Sigma S5261), G418 100 µg/mL (InvivoGen ant-gn-5, San Diego, CA, USA), and insulin 5 µg/mL (Sigma I9278). All supplements apart from HEPES and GlutaMax were purchased in Evercyte GmbH. Cells were seeded and maintained in 25 cm^2^ flask (TPP, Trasadingen, Switzerland, 90026) at 37 °C in a 5% CO_2_ atmosphere. Cells for experiments were used from passage 15 to 30.

### 2.3. Generation of Tubule-like Structures

RPTEC/TERT1 were seeded in µ-Slide 15 Well 3D (Ibidi, Gräfelfing, Germany, 81506) on the top of a hydrogel containing collagen I (Corning, NY, USA, 354236), Matrigel (Corning, NY, USA 354234), DMEM 5X (Sigma-Aldrich, St. Louis, MI, USA; D5523), NaOH 1N (Sigma-Aldrich, St. Louis, MI, USA; 221465), and MilliQ water. Different proportions of collagen I/Matrigel were used, 10/0, 90/10, 70/30, 50/50/and 0/10, respectively. RPTEC/TERT1 were also seeded on top of different coatings based on collagen I, Matrigel, and two different laminins (LN511-0202, LN521-02; BioLamina, Sundbyberg, Sweden). Cells were seeded on top of the hydrogel or the coating at 80.000 cells/cm^2^ density. For the generation of tubule-like structures, 5%MG was added to cell suspension. The culture media was changed every day and RPTEC/TERT1 grown for 7 days before nephrotoxicity assays. For the control condition, RPTEC/TERT1 were seeded in a 96-well plate. A schematic protocol can be found in Figure S1.

### 2.4. Cell Culture in the Microfluidic Device

RPTEC/TERT1 were seeded in a 6-channel device (μ-Slide VI 0.4; Ibidi, Gräfelfing, Germany). Cells were added within the channels at 225.000 cells/cm^2^ density. Once RPTEC/TERT1 reached confluence (3–4 days), shear stress was applied to the cell culture using a peristaltic pump (Ismatec, Masterflex Reglo Independent Channel Control, Avantor Fluid Handling, Gliwice, Polska) with two different shear stress regimes, 0.003 dyne/cm^2^ and 0.2 dyne/cm^2^ for 72 h at 37 °C. The lower flow rate was applied for maintenance purposes of the cell culture, in the absence of shear stress, and the second one to apply a physiological shear stress found in the kidney as reported in previous studies [8,25,26]. The experiments shown in Figure S3 were performed in a two channel fluidic device (BeFlow, BeOnChip, Zaragoza, Spain) using the same conditions as with the 6-channel device from Ibidi. For the control condition, RPTEC/TERT1 were seeded in a 96-well plate.

### 2.5. Immunofluorescence

Cells were fixed with 4% paraformaldehyde and permeabilized by 0.2% Triton X-100 (Sigma-Aldrich, Darmstadt, Germany, T8787) in PBS. Then, RPTEC/TERT1 were incubated with antibodies against acetylated α tubulin (1:200 dilution, sc-23950) and the tight junction protein *zonula occludens* (ZO-1, 1:200, Invitrogen, Waltham, MA, USA, 40-2200) overnight at 4 °C, followed by incubation with anti-rabbit-Alexa 488 (1:500, Invitrogen, Waltham, MA, USA, A11070) and anti-mouse-Alexa 546 (1:500, Molecular Probes, Eugene, OR, USA, A11030) for 1 h. Finally, DAPI nuclei staining (1:1000, Molecular Probes, Eugene, OR, USA, D3571) was added and incubated for 20 min. Confocal images were taken with a Nikon confocal microscope (Ti Eclipse EZ-C1,Nikon, Amstelveen, The Netherlands).

### 2.6. Quantitative Real-Time PCR

Total RNA extraction from RPTEC/TERT1 was carried out with Trizol (Invitrogen, Waltham, MA, USA, 15596026) following the supplier’s instructions. cDNA was synthetized from 500 ng of RNA using the commercial kit PrimeScript RT Master Mix (Takara, Saint-Germain-en-Laye, France, RR036A). qPCR was performed with the commercial kit Premix Ex Taq (Probe qPCR) (Takara, Saint-Germain-en-Laye, France, RR390L) using the real-time ViiA7 (Applied Biosystems, Waltham, Ma, USA)) according to the manufacturer’s protocol. Once the data on the cycles were obtained, the different conditions were analyzed with the 2^ddCt^ analysis method. RPLP0 gene was used as an endogenous control. All primer sequences are summarized in Table S1.

### 2.7. Cell Viability Assay

RPTEC/TERT1 were treated with CDDP and tacrolimus at 0, 15, 30, 50, 50, 100, and 300 μM and with daunorubicin at 0, 50, 100, 150, 150, 200, and 300 μM for 24 h. Cell culture media was renewed, and cells were kept in the incubator for 24 h. After treatments, a cell viability assay was performed using PrestoBlue (Life Technologies, Bleiswijk, The Netherlands, A13262). A plate reader (Biotek Synergy HT, Winooski, VT, USA) equipped with 528/90 excitation and 590/35 emission filters was used to read the fluorescence emitted by the PrestoBlue. Crystal violet was used as an additional method following the PrestoBlue assay in the kidney-on-a-chip model. Cells were washed with HBSS and fixed in cold absolute methanol for 10 min. Crystal violet (Sigma, Burlington, MA, USA, C0775) 0.1% in distilled water was added for 30 min. Cell culture was rinsed thoroughly with water and 10% acetic acid was added and kept in agitation (IKA MS 3 Digital, Barcelona, Spain) for 20 min. Finally, absorbance was measured at 590 nm. The data shown in the toxicity dose-response curves correspond to the relative data of the fluorescence (PrestoBlue) or absorbance (crystal violet) obtained in the plate reader.

### 2.8. Statistical Analysis

The data obtained were analyzed statistically with GraphPad Prism 6 software. The graphs show the mean with its standard deviation (SD). The statistical test used for the analysis of qPCR results was a two-way ANOVA. The statistical test used for the nephrotoxicity assays was the one-way nonparametric ANOVA with correction by the Tukey test, *p*-value < 0.05.

## 3. Results

### 3.1. Generation of Self-Assembled Tubule-like Structures

**Figure 1:**
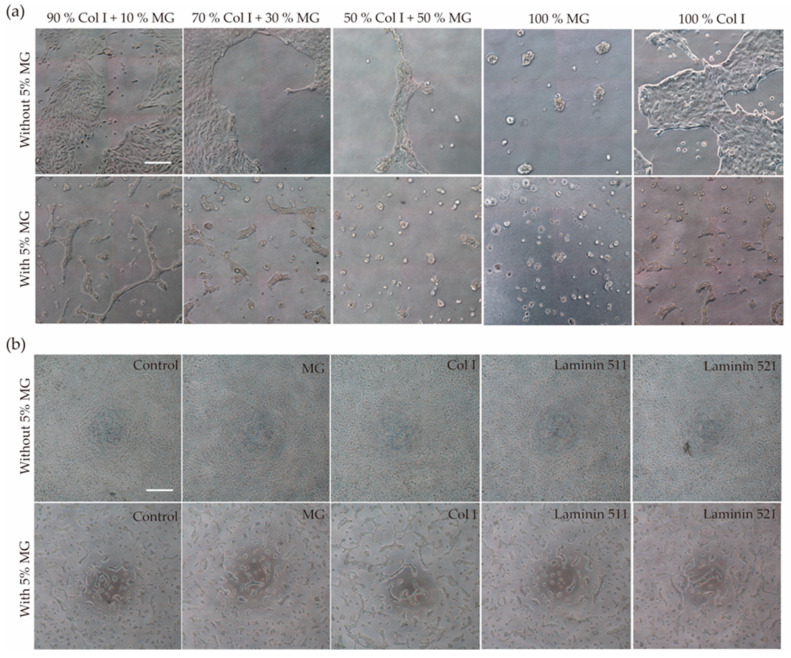
(**a**) Formation of self-organized structures. RPTEC/TERT1 were seeded on top of hydrogels with different proportions of collagen I and MG by adding 5% MG to the cell suspension before cell seeding or without 5% MG. Tubule-like structures were formed mostly in the 90% Col I + 10% MG hydrogel when compared with the other conditions. (**b**) RPTEC/TERT1 with 5% MG seeded on top of different coatings formed self-organized structures in all conditions, even in the control without coating. Scale bar 50 µm.

To determine the optimal conditions for the formation of three-dimensional structures from RPTEC/TERT1 cells, biological matrices were tested by mixing collagen I and Matrigel (MG) in different proportions or as the only component of the hydrogel. When cells were seeded on top of collagen I or MG hydrogel without 5% MG in the cell suspension, RPTEC/TERT1 failed to form tubule-like structures. In the 50/50 proportion, cell culture tended to create an elongated tubular structure (Figure 1a, upper row). When 5% MG was added to the cell suspension, tubule-like structures formed even in conditions where a low or no proportion of MG was added to the hydrogel mixture. On the other hand, when MG was the main component of the hydrogel or was in a higher proportion than collagen I, cyst-like structures were found (Figure 1a, bottom row).

To gain further insights into the mechanism underlying tubulogenesis caused by the addition of 5% MG to the cell suspension before cell seeding, two types of laminins were used. Laminins, abundant in MG, are basal membrane components that have been shown to play an important role in the morphogenesis of branching structures in renal cells in 3D cell culture [27]. RPTEC/TERT1 were resuspended in medium containing either 10% Laminin 511 or 521, but the cells did not form tubular structures (Figure S2a). These results suggest that other components of MG are involved in the formation of these self-organized structures with tubular morphology.

Once it was established that the presence of MG in the culture medium is the determining factor for tubule formation, it was necessary to see if this effect could be replicated in the absence of hydrogel by seeding the cell suspension with 5% MG on the plastic surface of a multi-well plate (2+D architecture). Self-organized structures with tubular shapes and cystic structures were formed in all conditions (Figure 1b).

**Figure 2:**
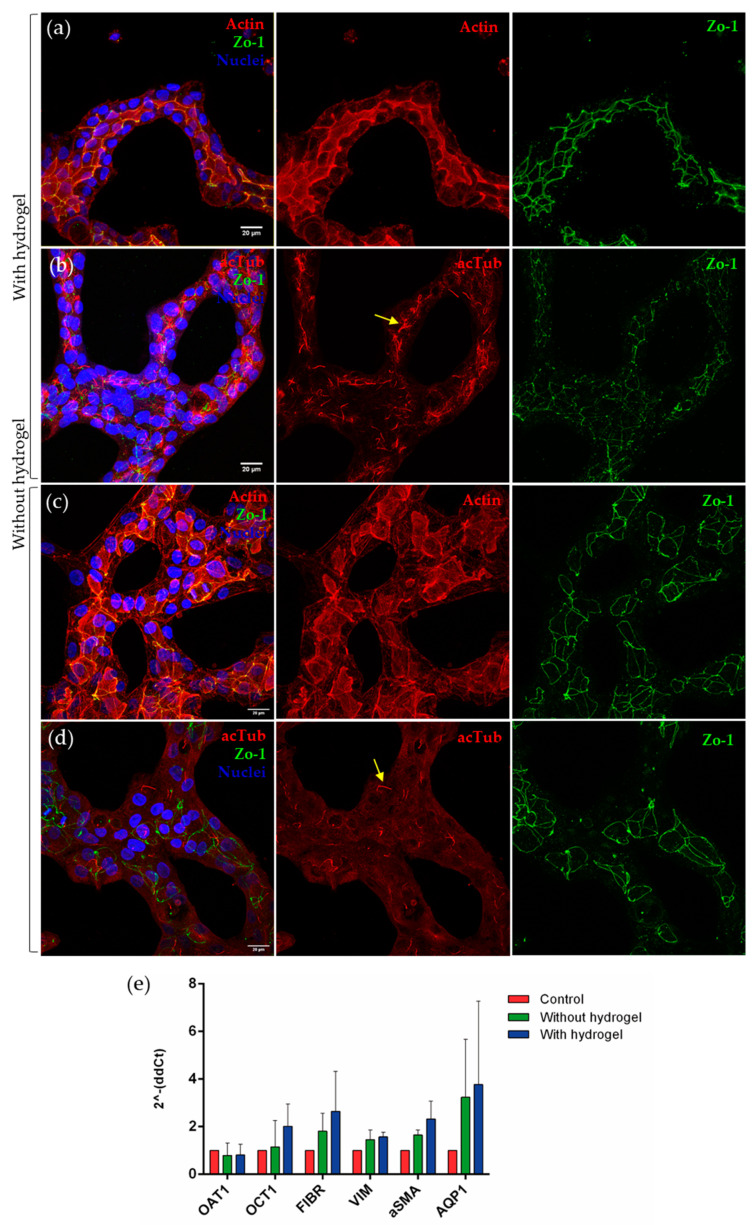
RPTEC/TERT1 with 5% MG on collagen I + Matrigel hydrogel (90:10). Confocal microscopy images show tubular formations of RPTEC/TERT1 on hydrogel (**a**,**b**) and without hydrogel (**c**,**d**). Acetylated tubulin is located in the primary cilium. The yellow arrows point to the presence of primary cilia in both conditions. The location of the actin in the apical region of the cells defines the lumen of the tubule-like structures. ZO-1 shows the tight junction between the proximal tubule cells. Scale bar: 20 µm. (**e**) The expression of transporters and dedifferentiation markers was analyzed in the three models, both in the control and in the tubules with or without gel. No significant differences were found between models, but increased expression of OCT1, fibrin, vimentin, α-SMA, and AQP1 was observed in tubulogenesis models. Two-way ANOVA, *n* = 3.

In order to characterize the degree of differentiation achieved in our 2.5D and 2+D cultures, different epithelial markers present in the proximal tubule cells were analyzed. RPTEC/TERT1 tubules were grown for seven days onto a hydrogel composed of 90% collagen I and 10% MG, or directly onto a collagen I-coated device, in the presence of 5% MG in the cell culture medium. Immunofluorescence was used to detect the following three markers: phalloidin to observe the arrangement of actin filaments, acetylated tubulin (acTub) to observe the primary cilium and cytoskeleton, and *zonula occludens* (ZO-1), a type of tight junction found in epithelial cells. In tubular polarized cells, the accumulation of actin beneath the apical membrane delineates the tubular lumen (Figure 2a). The presence of ZO-1 demonstrated the junction between the cells, delimiting their outline (Figure 2a,b). Acetylated tubulin was found predominantly in the primary cilium, a mechano-sensing organelle present in this cell type that also marks the polarization of these cells towards the tubular lumen because it is located in the apical part of the cells (Figure 2b).

On the other hand, when the same immunofluorescence technique was performed on RPTEC/TERT1 forming tubular structures in the absence of hydrogel, the same markers were observed (Figure 2c,d).

In both models, actin accumulated in the apical part of the cells, delimiting the lumen of tubular structures. ZO-1 was also present in both models, although staining for actin and acetylated tubulin was scattered, perhaps reflecting less differentiation (Figure 2d).

To quantitatively analyze the expression of renal-specific markers in the cell line used, several genes were studied (Figure 2e). A trend in the increase in the expression of OCT1 was observed in tubule-like structures with hydrogel when compared with the control condition. There was also a trend in the increase in AQP1 in both tubulogenesis models, and the same effect was found in markers related to epithelial-to-mesenchymal transition, such as fibronectin, vimentin, and α-SMA.

### 3.2. Development of a Kidney-on-a-Chip Model

**Figure 3:**
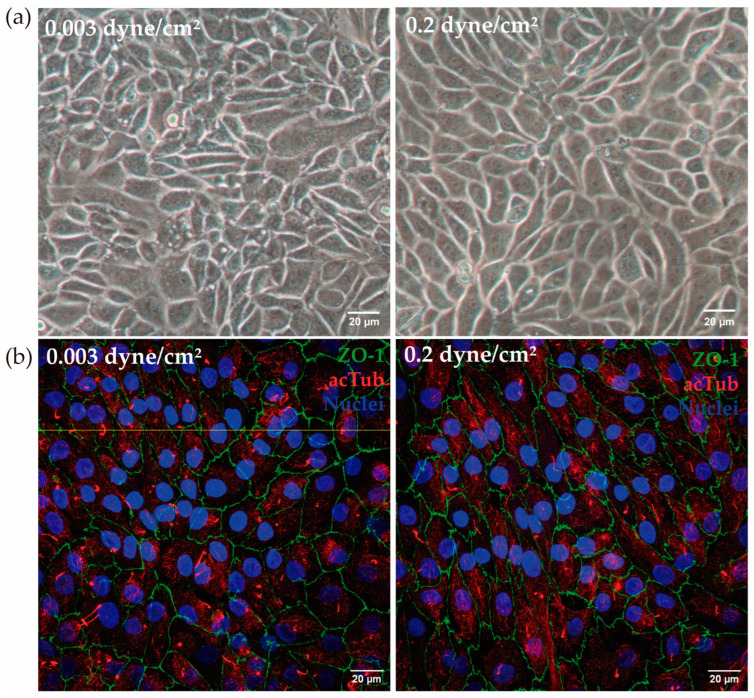
(**a**) RPTEC/TERT1 in the kidney-on-a-chip model. After 72 h at low flow (0.003 dyne/cm^2^) and high flow (0.2 dyne/cm^2^), cells maintained the monolayer in the channels of the device. (**b**) In a different experiment, the presence of ZO-1 (green) and acetylated tubulin (red) was analyzed. Both in low-flow and high flow conditions, the presence of ZO-1 and acetylated tubulin was confirmed. The yellow line indicates the height at which the Z-stack was made. Scale bar: 20 µm.

In the kidney-on-a-chip model under two different fluid flow conditions, the cell monolayer remained intact after 72 h and no changes in cell morphology between the two conditions were found (Figure 3a).

The cell membrane shows a characteristic serrated contour, as shown by the ZO-1 intercellular thigh junction stain. Moreover, no variation in cell height was observed when physiological shear stress was applied (Figure 3b).

**Figure 4:**
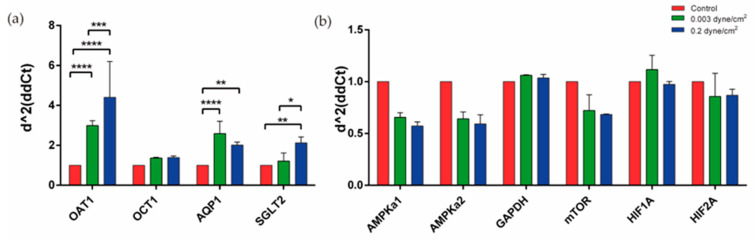
Expression of different markers present in the kidney-on-a-chip model. (**a**) Significant differences were found in the expression of OAT1, AQP1, and SGLT2, increasing their expression in flow conditions when compared with control. (**b**) Expression of different markers related to cellular metabolism were analyzed and no significant differences were found in flow conditions when compared with control. Two-way ANOVA, * *p* < 0.1, ** *p* < 0.01, *** *p* < 0.001, **** *p* < 0.0001. *n* = 2.

The next step was to analyze the expression of proximal tubule membrane transporters OAT1, OCT1, AQP1, and SGLT2 between the different conditions (Figure 4a). Expression of these transporters have been reported before in the literature in RPTEC/TERT1 [28]. Regarding OAT1 expression, significant differences were found between control and low flow (0.003 dyne/cm^2^), control and high flow (0.2 dyne/cm^2^), and between the two flow regimes, where its expression was increased compared to the control. AQP1 expression was also significantly increased in the two flow conditions compared to the control. Finally, the SGLT2 transporter also increased its expression in the two flow regimes compared to the control; significant differences were observed between the control and high-flow and between the two regimes. A decrease in both AMPKa catalytic subunits and mTOR expression was observed in both flow conditions compared to the control but no significant differences were found. Hypoxia markers HIF1A and HIF2A did not change the expression when compared to the control condition.

Similar results were reproduced in experiments carried out with the BeFlow microfluidic device (Figure S3). A significant decrease in AMPKa1, AMPka2, and mTOR expression was observed in both flow conditions compared to the control, as well as in HIF1A but only when comparing the low flow with the control condition. Regarding the transporters, the expression of OAT 1 and SGLT2 increased significantly when RPTEC/TERT1 were exposed to flow.

### 3.3. Nephrotoxicity Assays in Tubulogenesis and Kidney-on-Chip Models

In the tubulogenesis model, two nephrotoxic drugs, daunorubicin and cisplatin, were tested in three different conditions, control, 2+D structures, and 2.5D.

**Figure 5:**
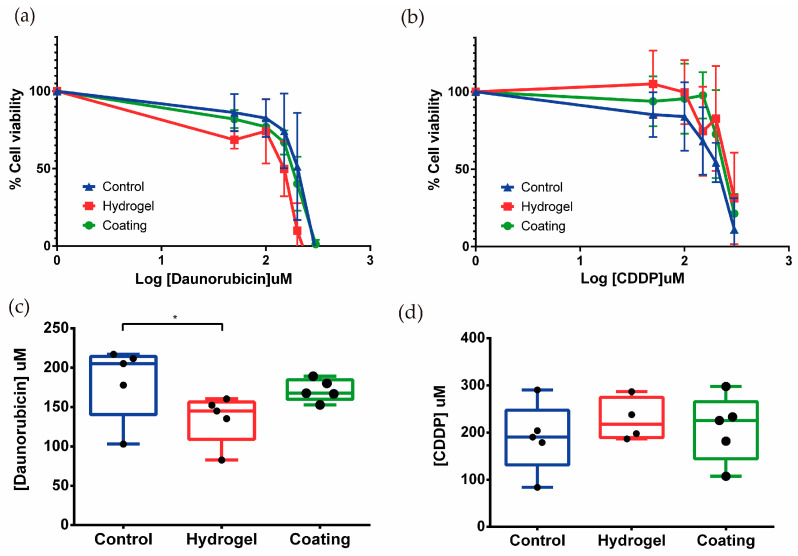
(**a**,**b**) Effect of daunorubicin and CDDP treatment on the viability of tubule-like structures. RPTEC/TERT1 on hydrogel showed lower drug resistance to daunorubicin, and the opposite happened when cells were exposed to CDDP. Mean ± SD, *n* = 5. (**c**,**d**) EC50 values were calculated for daunorubicin and CDDP. Tubules in the 2.5D model were significantly more sensitive when they were exposed to daunorubicin when compared to control. Mean with ± SD One-way ANOVA * *p* < 0.05. *n* = 5.

After treatment with daunorubicin or CDDP at different concentrations (0, 15, 30, 50, 100, and 300 μM) for 24 h, a dose-dependent decrease in cell viability was observed (Figure 5a,b). It is important to point out that the six-channel device used for these experiments allows the analysis of five different concentrations plus the control to be analyzed. In the device, RPTEC/TERT1 were exposed to the same shear stress to kinetically analyze the toxicity of the drugs.

The EC50 was calculated for both compounds. In the case of daunorubicin, the EC50 values for control, tubules on hydrogel (2.5D), and tubules on coating (2D) were 183.0 ± 47.2, 135.2 ± 30.7, and 171.3 ± 14.0 μM, respectively (Figure 5c). These results suggest that tubule-like structures on hydrogel would be more sensitive to daunorubicin treatment when compared to epithelial monolayers in the control condition.

Regarding CDDP treatment, no significant differences were observed between the EC50s of the three conditions, control, 2.5D, and 2D, whose values were 189.6 ± 73.6, 227.4 ± 45.4, and 209.2 ± 70.3 μM, respectively (Figure 5d). The results showed a tendency to increase resistance to CDDP when RPTEC/TERT1 recreated tubular structures. These results highlight the different phenotype unveiled by inducing tubulogenesis in RPTEC-TERT1 cells.

**Figure 6:**
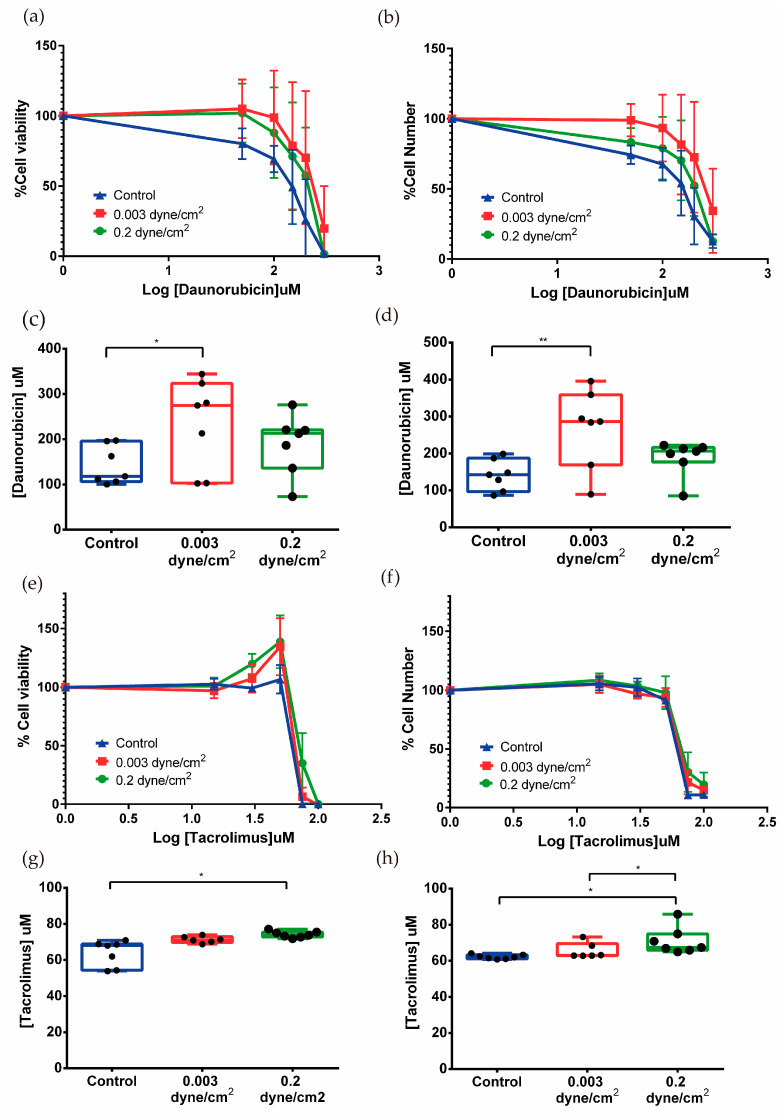
Effect of daunorubicin and tacrolimus in the kidney-on-a-chip model. The percentage of cell viability was measured by PrestoBlue (**a**,**e**) and the percentage of surviving cells with crystal violet (**b**,**f**). Mean ± SD, *n* = 7. EC50 was also measured using data from PrestoBlue (**c**,**g**) and crystal violet (**d**,**h**). Significant differences were found between cells exposed to fluid flow when compared to the control in cells treated with daunorubicin and tacrolimus. Mean with ± SD. One-way ANOVA. * *p* < 0.05, ** *p* < 0.005. *n* = 7.

For the kidney-on-a-chip, the daunorubicin and tacrolimus cytotoxic response was analyzed in RPTEC/TERT1 exposed for 72 h to the three conditions, control, low flow (0.003 dyne/cm^2^), and high flow (0.2 dyne/cm^2^) for 24 h. Here, dose–response toxicity was measured in two different ways, assessing cell viability (Figure 6a,c,e,g) and estimating the number of remaining cells (Figure 6b,d,f,h).

Both assays showed dose-dependent effects of daunorubicin on cell viability. Cells that had been subjected to flow appeared to be more resistant to daunorubicin as the curve was shifted to the right with respect to the control curve. The EC50 was calculated from cell viability and cell number data. The cell viability results for control, low flow, and high flow conditions were 141.8 ± 42.6, 234.4 ± 99.0, and 189.330 ± 66.165 µM, respectively (Figure 6c). For cell number, similar data were obtained, which were 141.2 ± 42.0, 268.287 ± 106.0, and 188.5 ± 47.8 µM (Figure 6d). The data suggest that in the conditions where flow has been applied, cells become more resistant to daunorubicin since the EC50 is higher in both cases with respect to the static control.

Exposure of RPTEC/TERT1 to flow conditions induces a phenotypic change in their response to increasing doses of tacrolimus. For doses ranging from 15 to 50 µM, an increase in cell viability with an equal number of cells was observed, suggesting tacrolimus at low doses stimulates metabolism in flow-conditioned RPTEC cells. Higher doses caused a sharp decrease in cell viability and surviving cells (Figure 6e,f).

EC50 values of tacrolimus for cell viability in the three conditions (control, low flow, and high flow) were 63.8 ± 7.2, 71.3 ± 1.8, and 74.2 ± 1.8 µM, respectively (Figure 6g). The EC50 values obtained for surviving cell numbers show a similar moderate but significant increase: control 62.2 ± 1.2, low flow 65.6 ± 4.3, and high flow 71.0 ± 7.4 µM (Figure 6h). Here, the application of physiological shear stress increased the resistance to drug exposure when compared to the control condition. On the other hand, it seems that flow rate also has an influence since there are significant differences between the EC50 of low flow and high flow, the latter corresponding to the model where physiological shear stress is applied (Figure 6h).

## 4. Discussion

The application of new in vitro kidney models that attempt to biomimic what occurs in native tissue in the human body has proven useful for drug nephrotoxicity testing [29]. These models have been developed over the years with the aim of representing renal physiology in the human body, to overcome issues with traditional cell culture or animal models [13,30,31,32,33]. However, these new approaches, being in constant development, are difficult to implement in research due to both their complexity and their high economic cost. Because of this, it is necessary to standardize and define the parameters of emerging models to obtain data that can be useful in the development of new drugs [2]. In this sense, we have developed two models that attempt to biomimic the cellular phenotype of renal proximal tubule cells, in order to observe the response of these models to different drugs such as cisplatin, daunorubicin, and tacrolimus, which are known for their nephrotoxic potential [34,35]. Our study provides useful insights into the relevance of phenotypic changes induced by the mechano-chemical stimulus provided by the hydrogel or a mechanical stimulus such as shear stress.

In the tubulogenesis model, there was a significant difference in daunorubicin EC50 between cells that formed tubular structures on a hydrogel and the control. RPTEC/TERT1 were shown to be more sensitive to the drug when they formed such structures on the hydrogel. However, in the kidney-on-a-chip model, cells that were subjected to physiological shear stress showed a tendency to be more resistant to the drug. Daunorubicin is an anthracycline like doxorubicin, and they share the same mechanism of action. In previous studies of in vitro kidney models, testing doxorubicin, where tubular structures embedded in a biological matrix were formed, it was observed that 3D cells were more sensitive to doxorubicin compared to 2D culture [17]. This is similar to our findings with daunorubicin, which had not been tested in in vitro studies before. In the kidney-on-a-chip model, however, cells exposed to physiological shear stress become more resistant to daunorubicin compared to cells under static conditions (control). These apparently paradoxical results highlight the relevance of the experimental model in the final result. It is worth mentioning that both models differ in the drug access route (luminal for the fluidic model and basolateral in the 2+D tubules). A detailed mechanistic explanation was outside the scope of the current study.

The tubulogenesis model was developed to provide a straightforward and reproducible protocol for analyzing various drugs, suitable for most laboratories. Matrigel and collagen I were used for the development of this model, both of which are composed by biological components present in the ECM, commercially available and accessible due to their standardized use in the development of three-dimensional models [18,36]. However, this method has limitations when assessing cell viability. Specifically, when cells form tubules on a hydrogel, the hydrogels can detach from the wells during the assay due to the multiple washes required. More importantly, the reagent diffuses into the hydrogel, interfering with the fluorescence readings. This artefact was avoided with the hydrogel-free tubulogenesis model, which is a simpler and more affordable model to implement in the laboratory. Regarding the kidney-on-a-chip model, the EC50 data for the two flow conditions indicate that RPTEC/TERT1 cells exposed to shear stress are more resistant to the drug compared to the static control. This effect is statistically significant between the control and the cells exposed to a physiological shear stress of 0.2 dyne/cm^2^. Additionally, significant differences were observed between the two flow conditions, with a higher EC50 in the model with physiological shear stress when cells were treated with tacrolimus. This indicates that the protective effect is related to exposure to this physiological-specific stimulus. In the kidney-on-a-chip, when the analysis of tacrolimus toxicity was performed using the PrestoBlue assay, two different peaks were observed in cell viability at 30 and 50 µM in the two fluid flow conditions when compared to the control. This effect was not observed when the crystal violet assay was performed. At sub-toxic concentrations as 30 and 50 µM, tacrolimus activates cell metabolism and this effect was visible in the PrestoBlue assay. The valuable point is that our model has been able to detect it since it has the sensitivity to show those changes.

Kidney models based on new technologies have evolved over the years and more complex models have also been developed, recreating a tubule-like structure in a commercial microfluidic device (OrganoPlate, Mimetas, Leiden, The Netherlands) for nephrotoxicity and kidney transport assays [11]. Microfluidics have also been combined with the culture of kidney organoids inside a millifluidic device, applying flow over their surface to see how these organoids responded to the shear stress produced by the culture medium. The results showed an increase in vascularization and an increase in the number of cells from both the tubular and glomerular parts of the nephron [37]. While it is true that more complex models are closer to what occurs in renal tissue in vivo, they also have their limitations. They are difficult to implement in the laboratory, require highly qualified skills, and are expensive. For those reasons, this type of methodology is not used systematically on a large scale in drug cytotoxicity tests. In this study, the main goal was to make these techniques more accessible and user-friendly to generate standardized models for drug testing.

Both the tubulogenesis model and the kidney-on-a-chip model developed here still face several limitations. Despite recreating a more physiological microenvironment, epithelial–mesenchymal transition (EMT) markers are still observed, indicating incomplete differentiation. Additionally, the tubule-like structures generated on top of hydrogels are not perfused, limiting their functionality. On the other side, in the kidney-on-a-chip model, the cell cultures are perfused, but RPTEC/TERT1 grow as a monolayer on a rigid surface, which means that a completely physiological model was not fully achieved.

It is also important to highlight the different responses obtained with different drugs, not in terms of what changes they induce, but in terms of the different behaviors between the tubulogenesis and the kidney-on-a-chip models. For instance, daunorubicin performs poorly in control conditions when compared to the 2.5D model and the kidney-on-a-chip model, but reproducibility is better in 2.5D cultures. These limitations highlight the need for further optimization to better replicate the complex in vivo environment.

We have established several advanced culture models that respond differently to nephrotoxic agents compared to conventional cell cultures. Our findings support the standardized use of these models for nephrotoxicity studies due to their reproducibility and ease of integration into routine laboratory practices. This is relevant not only for well-known nephrotoxic agents but also for investigating new compounds. Examining the response to a broader range of nephrotoxic drugs using these new models is crucial for comprehensive toxicity assessments and better understanding the models’ versatility.

## Data Availability

The raw data supporting the conclusions of this article will be made available by the authors on reasonable request.

## Supplementary Materials

The following supporting information can be downloaded at: https://www.mdpi.com/article/10.3390/biomimetics9070446/s1, Figure S1. RPTEC/TERT1 cell culture in 2,5 D model. Before cell seeding, different hydrogels or coatings were added at the bottom of the wells. Then, RPTEC/TERT1 were seeded directly on top of the hydrogels or coatings or adding 5% Matrigel to the cell suspension. Created with BioRender.com. Figure S2. RPTEC/TERT1 with 10% Laminin on top of hydrogel. Cells did not form tubule-like structures when they were seeded with 10% of laminin 511, laminin 521 or a mixture of both in the cell suspension (a–c) in comparison with the positive condition (a). Figure S3. RPTEC/TERT were seeded in a different commercial microfluidic device (Be-flow, BeOnChip). (a,b) After 72 hours at low flow (0.03 dyne/cm^2^) and high flow (0.2 dyne/cm^2^), the presence of ZO-1 (green) and acetylated tubulin (red) both in low flow and high flow conditions was confirmed. The yellow line indicates the height at which the Z-stack was made. Yellow arrows indicate the presence of primary cilia. Scale bar: 20µm. (c) Expression of different markers present in the kidney-on-a-chip model in the Be-flow device. Significant differences were found in the expression of OAT1 and SGLT2, increasing their expression in flow conditions when compared with control. (d) Expression of different markers related to cellular metabolism were analyzed and significant differences were found in all markers between flow conditions and the control condition. Two-way ANOVA, * *p* < 0.1, ** *p* < 0.01, *** *p* < 0.001, *n* = 3. Table S1. Markers used in qPCR for the tubulogenesis model and for the kidney-on-a-chip model.
