# Supplementary material for: Advanced Kidney Models In Vitro Using the Established Cell Line Renal Proximal Tubular Epithelial/Telomerase Reverse Transcriptase1 for Nephrotoxicity Assays
Source: Biomimetics (Basel). 2024 Jul 22;9(7):446. doi: 10.3390/biomimetics9070446 (PMC11275192; doi:10.3390/biomimetics9070446)
Supplement: Supplementary file 1 [file biomimetics-09-00446-s001.zip › biomimetics-3075948-supplementary.pdf]

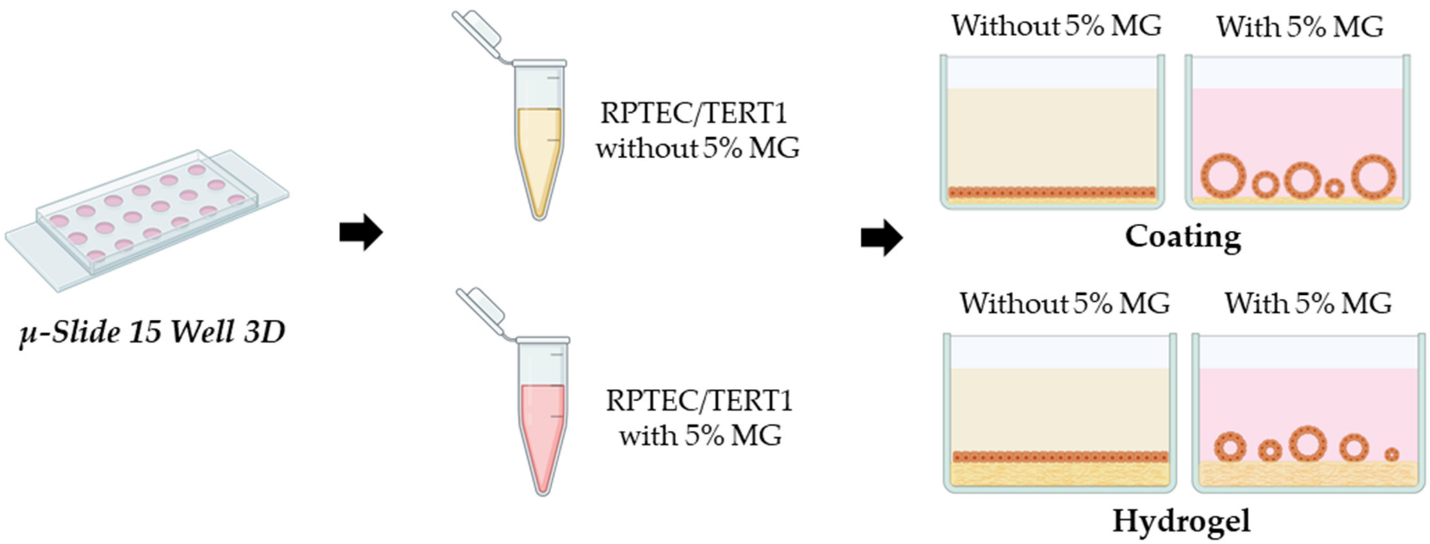

Figure S1. RPTEC/TERT1 cell culture in 2,5 D model. Before cell seeding, different hydrogels or coatings were added at the bottom of the wells. Then, RPTEC/TERT1 were seeded directly on top of the hydrogels or coatings or adding 5% Matrigel to the cell suspension. Created with BioRender.com

Table S1. Markers used in qPCR for the tubulogenesis model and for the kidney-on-a-chip model.

Gene	RefSeq Number	Sequence
RPLP0	NM_001002	Probe: 5'-/56-FAM/CCCTGTCTT/ZEN/CCCTGGGCATCAC/3IABkFQ/-3'<br>Primer 1: 5'-TCGTCTTTAAACCCTGCGTG-3'<br>Primer 2: 5'-TGTCTGCTCCCACAATGAAAC-3'
ACTA2 (α-SMA)	NM_001141945	Probe: 5'-/56-FAM/AGACCCTGT/ZEN/TCCAGCCATCCTTC/3AIBkFQ/-3'<br>Primer 1: 5'-AGAGTTACGAGTTGCCTGATG-3'<br>Primer 2: 5'-CTGTTGTAGGTGGTTTCATGA-3'
FANK 1(Fibronectin)	NM_145235	Probe:5'-/56-FAM/TGCCACTTC/ZEN/CATTCTTCAGATTCACGT/3IABkFQ/-3'<br>Primer 1: 5'-TTGGCTTTACCGCTCTGATG-3'<br>Primer 2: 5'-CACGCCAGCATTAGACTGT-3'
VMAC (Vimentin)	NM_001017921	Probe: 5'/56-FAM/CAAGGACCG/ZEN/TGAGATTGCCACACT/3IABkFQ/-3'<br>Primer 1: 5'-CCGCCCTAGACGAACTG-3'<br>Primer 2: 5'-CTGCAACTGCCTAATGAGC-3'
SLC22A1 (OCT1)	NM_153187	Probe: 5'/56-FAM/AGCGCACCT/ZEN/TCATCCTGATGTACC/3IABkFQ/-3'<br>Primer 1: 5'-CCTTCATTTGCAGACCTGTTC-3'<br>Primer 2: 5'-TGATAGAGCACAGAGTCCGT-3'
SLC22A6 (OAT1)	NM_153278	Probe: 5'-/56-FAM/TCCATGACC/ZEN/AGCCCATAGTATGCAAAG/3IABkFQ/-3'<br>Primer 1: 5'-CTCTTCCTCTGCCTCCCAT-3'<br>Primer 2: 5'-ACAGCACCAAAGATCACCTG-3'
AQP1	NM_001185060	Probe: 5'-/56-FAM/CAGCCAGTG/ZEN/TAGTCAATAGCCAGGAG/3IABkFQ/-3'<br>Primer 1: 5'-CCTCTCTGTAGCCCTTGGA-3'<br>Primer 2: 5'-CCCACCCAGAAAATCCAGT-3'
SLC5A2 (SGLT2)	NM_003041(1)	Probe: 5'-/56-FAM/CGAAGGTCT/ZEN/GTACCGTGTCCGTG/3IABkFQ/-3'<br>Primer 1: 5'-CATCTATGCCTCCGTCATCG-3'<br>Primer 2: 5'-GGCGTAACCCATGAGGATG-3'
PRKAA1 (AMPKa1)	NM_206907	Probe: 5'-/56-FAM/TGTTTGCCA/ZEN/ACCTTCACTTTGCCG/3IABkFQ/-3'<br>Primer 1: 5'-AAGATCGGCCACTACATTCTG-3'<br>Primer 2: 5'-ACAGCTACTTTATGCCCAGTC-3'
		Probe: 5'-/56-FAM/CCTGCATAC/ZEN/AATCTGCCTGAGATGACT/3IABkfq/-3'

AMPKa2	NM_006252	Primer 1: 5'-GTGAATTTCTGAGAACTAGTTGCG-3'<br>Primer 2: 5'-AAGATAACACCACAGCTCCAG-3'
GAPDH	NM_002046	Probe: 5'-/56-FAM/AAGGTCGGA/ZEN/GTCAACGGATTTGGTC/3IABkFQ/-3'<br>Primer 1: 5'-ACATCGCTCAGACACCATG-3'<br>Primer 2: 5'-TGTAGTTGAGGTCAATGAAGGG-3'
mTOR	NM_004958	Probe: 5'-/56 FAM/CCATTGGAA/ZEN/TGAAAATTTGGTACTTCTTCCCC/3IABkFQ/-3'<br>Primer 1: 5'-GACCAGAGCCCAGAACTG-3'<br>Primer 2: 5'-ATGATTGATTCGGTGTCGCA-3'
HIF1A	NM_181054	Probe: 5'-/56-FAM/TGGCAAGCA/ZEN/TCCTGTACTGTCCTG/3IABkFQ/-3'<br>Primer 1: 5'-CTCTGATCATCTGACCAAAACTCA-3'<br>Primer 2: 5'-CAACCCAGACATATCCACCTC-3'
EPAS1 (HIF2A)	NM_001430	Probe: 5'-/56-FAM/AGAGTCACC/ZEN/AGAACTTGTGCACCAA/3IABkFQ/-3'<br>Primer 1: 5'-AGCCTATGAATTCTACCATGCG-3'<br>Primer 2: 5'-CTTTGCGAGCATCCGGTA-3'

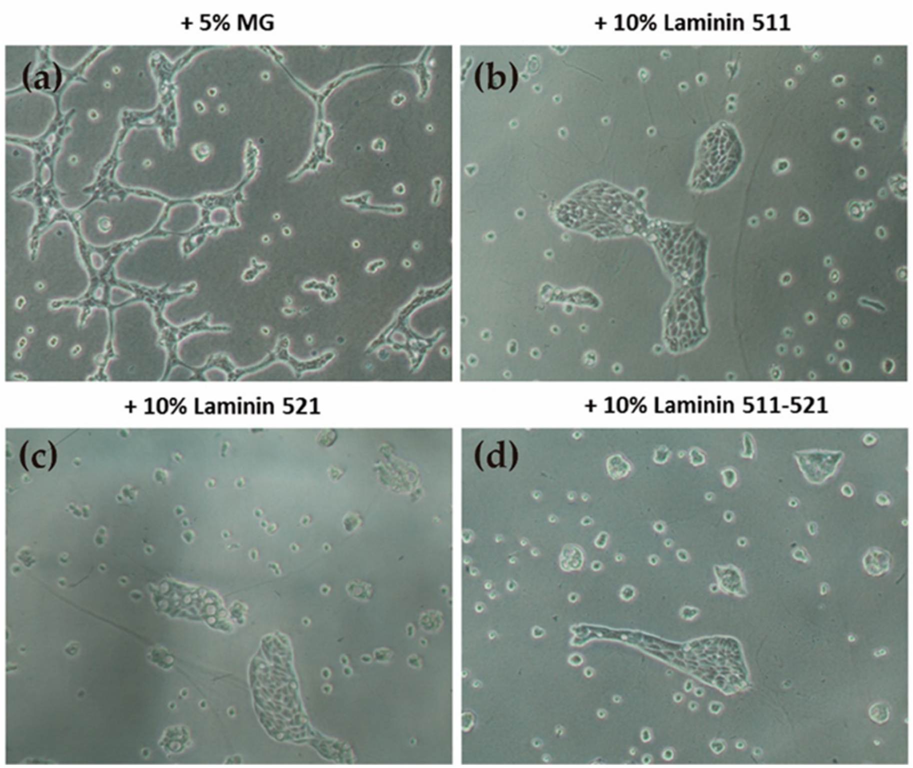

Figure S2. RPTEC/TERT1 with 10% Laminin on top of hydrogel. Cells did not form tubule-like structures when they were seeded with 10% of laminin 511, laminin 521 or a mixture of both in the cell suspension (a, b, c) in comparison with the positive condition (a).

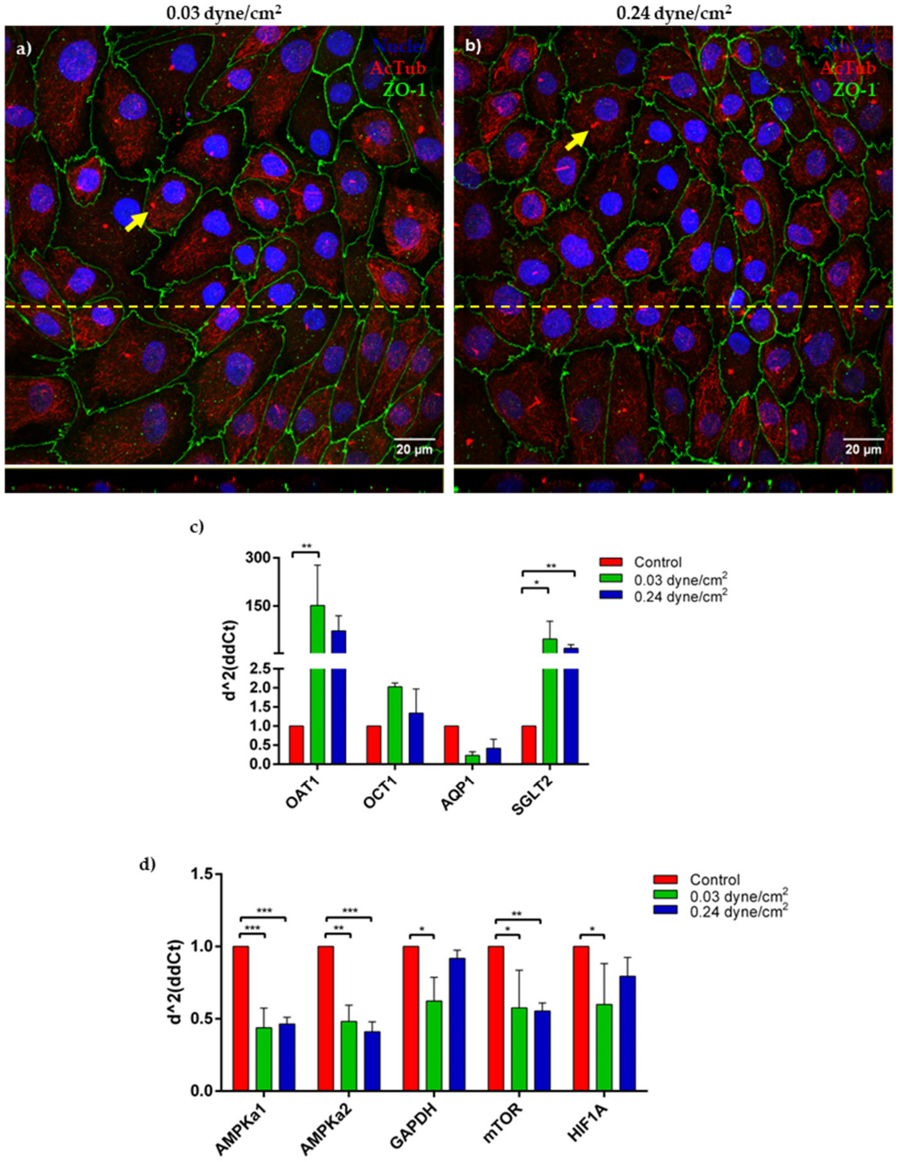
0.03 dyne/cm2
0.24 dyne/cm2
a)
b)
AcTub
ZO-1
20 µm
c)
Control
0.03 dyne/cm2
0.24 dyne/cm2
d^2(ddCt)
OAT1
OCT1
AQP1
SGLT2
d)
AMPKa1
AMPKa2
GAPDH
mTOR
HIF1A

Figure S3. RPTEC/TERT were seeded in a different commercial microfluidic device (Be-flow, BeOnChip). (a,b) After 72 hours at low flow (0.03 dyne/cm^2) and high flow (0.2 dyne/cm^2), the presence of ZO-1 (green) and acetylated tubulin (red) both in low flow and high flow conditions was confirmed. The yellow line indicates the height at which the Z-stack was made. Yellow arrows indicate the presence of primary cilia. Scale bar: 20μm . (c) Expression of different markers present in the kidney-on-a-chip model in the Be-flow device. Significant differences were found in the expression of OAT1 and SGLT2, increasing their expression in flow conditions when compared with control. (d) Expression of different markers related to cellular metabolism were analyzed and significant differences were found in all markers between flow conditions and the control condition. Two-way ANOVA, * $p < 0.1$, ** $p < 0.01$, ***$p<0.001$, n=3.
